# Microscopic Colitis and Celiac Disease: Sharing More than a Diagnostic Overlap

**DOI:** 10.3390/nu16142233

**Published:** 2024-07-11

**Authors:** Ana María González-Castro, Fernando Fernández-Bañares, Yamile Zabana, Georgina Farago-Pérez, Jonathan Ortega-Barrionuevo, Elba Expósito, Danila Guagnozzi

**Affiliations:** 1 Translational Mucosal Immunology Laboratory, Vall d’Hebron Institut de Recerca, 08035 Barcelona, Spain; amgonalez75@gmail.com (A.M.G.-C.); elbaexposito@vhir.org (E.E.); 2 Neuro-Immuno-Gastroenterology Laboratory, Vall d’Hebron Institut de Recerca, 08035 Barcelona, Spain; 3 Gastroenterology Department, University Hospital Mútua Terrassa, 08221 Terrassa, Spainyzabana@mutuaterrassa.es (Y.Z.); 4 Centro de Investigación Biomédica en Red en Enfermedades Hepáticas y Digestivas (CIBERehd, Instituto Carlos III), 28029 Madrid, Spain; 5 Gastroenterology Department, University Hospital Vall d’Hebron, 08035 Barcelona, Spain

**Keywords:** microscopic colitis, celiac disease, autoimmunity

## Abstract

Microscopic colitis (MC) is an emergent group of chronic inflammatory diseases of the colon, and celiac disease (CD) is a chronic gluten-induced immune-mediated enteropathy affecting the small bowel. We performed a narrative review to provide an overview regarding the relationship between both disorders, analyzing the most recent studies published at the epidemiological, clinical and pathophysiological levels. In fact, MC and CD are concomitantly prevalent in approximately 6% of the cases, mainly in the subset of refractory patients. Thus, physicians should screen refractory patients with CD against MC and vice versa. Both disorders share more than a simple epidemiological association, being multifactorial diseases involving innate and adaptive immune responses to known or unknown luminal factors based on a rather common genetic ground. Moreover, autoimmunity is a shared characteristic between the patients with MC and those with CD, with autoimmunity in the latter being quite well-established. Furthermore, CD and MC share some common clinical symptoms and risk factors and overlap with other gastrointestinal diseases, but some differences exist between both disorders. More studies are therefore needed to better understand the complex mechanisms involving the common pathogenetic ground contributing to the CD and MC epidemiological association.

## 1. Introduction

Microscopic colitis (MC) is an emergent group of chronic inflammatory diseases of the colon defined by the presence of chronic or intermittent watery diarrhea, endoscopically normal (or near normal) colonoscopy and specific histopathological features that define the principal subtypes of the disease, collagenous colitis (CC) and lymphocytic colitis (LC) [1]. Both MC subtypes are characterized by a significant increase in lamina propria inflammatory infiltrate and mild degenerative and/or regenerative epithelial damage. In particular, CC is defined by an increased subepithelial collagen band thickness (≥10 μm) and LC by a significant increase in the number of intraepithelial lymphocytes (IELs) (≥20 per 100 surface epithelial cells) [1]. Since the first description in 1976, MC has since been recognized as a worldwide common cause of chronic diarrhea with watery stools, showing an increasing incidence over time, now reaching 11.4/100,000 habitants/year (95% CI, 9.2–13.6, *I*^2^ = 99.7%) [1]. The pooled overall prevalence of MC is estimated to be 119 per 100,000 persons (95% CI: 73–166) [1], being more frequent in women over 60 years old and significantly impacting their health-related quality of life [1]. Furthermore, it is relevant to highlight that the diagnosis of MC is based on a complete colonoscopy with multiple biopsies since there is no biomarker available for MC diagnosis and/or follow-up. Moreover, oral budesonide is the first-choice treatment for these patients, as well as being an immunosuppressive treatment or biological agent in MC budesonide-refractory disease and other inflammatory bowel diseases [1]. 

On the other hand, celiac disease (CD) is a chronic gluten-induced immune-mediated enteropathy affecting the small bowel that develops in predisposed individuals [2]. It has a long history, first being described during the time of the ancient Greeks; however, there have been marked increases in the prevalence and incidence of this disease worldwide over the last 50 years. In fact, the global prevalence of CD is estimated to be between 0.7% and 1.4% of the general population [3]. It varies according to ethnicity and geography, with a higher prevalence in Caucasian and Nordic countries [4]. In Europe, the seroprevalence rate is estimated to be 1.4%, while the prevalence of CD diagnosed by biopsy is 0.7% [4]. The diagnosis of CD is characterized by a concordance between the clinical symptoms, positive serology (autoantibodies to anti-tissue transglutaminase, anti-endomysial and/or anti-deamidated gliadin peptide antibodies) and characteristic histopathological changes regarding the duodenal histology (villous atrophy and intraepithelial lymphocytosis) [2]. While a duodenal biopsy is still required to confirm the diagnosis of CD in adult patients, there is a growing body of evidence supporting the high accuracy of a no-biopsy approach in select cases due to the existence of useful non-invasive biomarkers for CD. Moreover, unlike patients with MC, the diet approach represents the main treatment for patients with CD, and a gluten-free diet (GFD) is the first-line treatment for these patients [2]. 

The present narrative review aims to provide an overview regarding the relationship between MC and CD by analyzing the most recent studies published at the epidemiological, clinical and pathophysiological levels.

## 2. Materials and Methods

We performed a review of the literature using the PubMEd, Web of Science and Cochrane Library databases in order to identify all articles written in the English language regarding the relationship between MC and CD without any restrictions for country or publication date. Search terms included the following words: CC, LC, MC and CD, GFD and HLA-DQ haplotypes. A backward search of the references of the articles also identified other relevant articles, while a forward search found newer articles included in the original cited paper.

## 3. The Epidemiological Association between MC and CD

Previous prospective and retrospective studies have shown a significant association between the two conditions, with a 50- to 70-fold increased risk of CD in MC patients compared to the general population [5]. Moreover, MC and CD are concomitantly prevalent in approximately 6% of cases [6,7], mainly in the subset of refractory patients. Two meta-analyses were published in 2022 investigating the epidemiological association between both disorders. 

A meta-analysis by Nimri F.M. et al. [6] included twenty-six studies published from 1997 to 2021, with a total of 22,571 MC cases and 3593 CD cases. CD was significantly associated with MC with a pooled odds ratio (OR) 8.276 (95% CI: 5.888–11.632, *p* < 0.001). The pooled event rate for CD was 6.1% (95% CI: 3.9–9.5%, *p* < 0.001). Specifically, the event rates were 5.2% (95% CI: 2.2–12.1%) for CC patients and 6.3% (95% CI: 3.4–11.5%, *p* < 0.001) for LC patients [6]. A case–control study by Wildt S. et al. [8] was conducted in Denmark and included the largest number of cases in this meta-analysis, with over 15,500 participants. The pooled event rate for the MC in patients with CD was 6.2% (95%CI: 4.1–9.2%, *p* < 0.001) [6]. Otherwise, for the two subtypes of MC, the pooled event rate was 1.6% (95%CI: 0.7–3.5%, *p* < 0.001) in the CC patients and 4.3% (95%CI: 3.1–5.9%, *p* < 0.001) in the LC patients [6]. However, according to a recent prospective cross-sectional analytical study, the prevalence of CD was 1.1% in the MC patients [9]. Moreover, there were no differences in the autoantibody titers between the MC patients responsive and resistant to treatment [9]. 

The meta-analysis by Aziz M. et al. [7] specifically evaluated the prevalence of MC in refractory CD patients and the prevalence of CD in refractory MC patients, including a total of twenty-six studies published from 1992 to 2019. The pooled prevalence of MC was 4.5% (2.6–6.3%, *I*^2^ = 78.8%) in patients diagnosed with refractory CD, including five studies with a total of 2589 patients [7]. Only the study by Leeds J.S. et al. [10] compared the prevalence of MC in refractory CD and a control population, observing that the prevalence of MC was higher in the refractory CD cases (1.6%) compared to the control population (0%). Otherwise, the pooled prevalence of CD was 6.7% (5.2–8.1%, *I*^2^ = 77.5%) in those patients previously diagnosed with MC, including the analysis of twenty-one studies [7]. Moreover, a meta-regression analysis showed an increasing prevalence of CD in refractory MC with diarrhea (Q = 0.05, *p* = 0.03) [7]. Therefore, it was calculated that the prevalence of CD was eight times (OR 8.12, 95% CI 4.92–13.41, *I*^2^ = 13.8%, *p* < 0.001) more likely in patients with refractory MC compared with the control group, as shown in five studies [7].

Recently, a nationwide population-based study was published in Sweden, including 45,138 CD patients and 223,149 MC patients. The MC incidence rate of 86.6 per 100,000 person-years (95%CI: 78.6–94.5) was described in the CD patients compared to 7.5 per 100,000 person-years (95%CI: 6.5–8.6) in the control population [11]. Although the risk of developing MC was highest in the first year, it remained elevated after 10 years of follow-up (aHR 35.2, 95%CI: 20.1–61.6 and aHR 8.1, 95%CI: 6.0–10.9), suggesting an independent association between CD and MC in terms of the surveillance bias and lymphocytic infiltration related to an active and untreated CD [11]. An earlier age in CD patients diagnosed with MC of almost 10 years was observed compared to the reference individuals. Considering the two subtypes of MC separately, the adjusted hazard ratio was 10.2 (95%CI: 7.7–13.6) for CC and 12.4 (95%CI: 10.0–15.3) for LC, observing that the LC subtype was the most common subtype associated with CD [11]. Finally, they found a 116-fold increased risk for MC (95% CI: 9.8–13.8) in the patients with CD [11]. Moreover, another important finding of this study was the excess risk that persisted in the sibling analysis, suggesting that CD and MC share genetics or early environmental factors that may play a role in the pathogenesis of both entities [11]. Furthermore, in another nationwide population-based study performed using the database of International Business Machine Explorys from the USA, the patients with CD were found to have one of the highest probabilities of being associated with MC (OR 22.5) among medical comorbidities [12].

Considering the patients’ characteristics with both concomitant diseases, a follow-up study showed that 4.3% of the patients with CD were diagnosed with MC after a prospective follow-up over 25 years, being older and with greater duodenal atrophy [13]. This observation could be an indication that MC is more commonly diagnosed in CD (64%) than the other way round (25%). Moreover, CC patients with CD had an earlier onset of their colitis, and the majority of the patients with CC and concomitant CD were smokers [14]. Finally, in a recent study in Saudi Arabia that performed multivariate analyses using the National Inpatient Sample (NIS) database, the presence of CD in the patients with MC was found to be a significant independent variable for in-hospital mortality (OR: 3.37, 95% CI: 1.32–8.60, *p* = 0.011), without an impact on the mean hospital stay [15]. 

Moreover, the relationship between MC and mild duodenal damage is still not well-established. In the first study published on the prevalence of duodenal Marsh type I lesion in MC, the authors observed that three-quarters of the MC patients with Marsh 1 duodenal histology and HLA-DQ2 expression responded to GFD, observing a rare presence of Marsh Type III CD lesions in the MC patients [16]. Subsequently, a strong association was observed between Marsh–Oberhuber type I duodenal injury and MC, especially LC [17]. In fact, 52.1% of the patients that underwent a colonoscopy with multiple biopsies had MC, suggesting the possible existence of “microscopic enterocolitis” and especially “Lymphocytic enterocolitis”, which could involve the entire gastrointestinal tract [17]. Further studies are needed to better understand the relationship between mild duodenal injury and MC subtypes.

However, it is important to bear in mind that there are some limitations in the studies published investigating the epidemiological relationship between MC and CD. In fact, adequately powered cohort studies representative of different countries are lacking. Moreover, there is an increased heterogeneity regarding the diagnostic methodology used for the diagnosis of MC and CD over time, especially if we consider the MC group. In fact, in some studies, the MC diagnosis was mainly based on non-validated histopathological diagnoses or on both non-clinical and non-histopathological criteria, especially for retrospective studies introducing biases and reliance on potentially inaccurate code or diagnosis information. Furthermore, the lack of stratified data of some studies with respect to the MC subtypes was observed in several studies. Finally, it is important to stress that most of the studies in CD and some in MC patients were performed in refractory cases, thus limiting the extrapolation of the results to non-refractory cases.

## 4. Common Ground and Sticking Points in Clinical Aspects between CD and MC

### 4.1. Risk Factors

CD is known to affect all age groups, with more than 70% of the new cases diagnosed over the age of 20 years; however, MC is more prevalent in advanced-age patients (more than 60 years old), with the diagnosis of the disease in much younger patients observed only in one-quarter of the cases [18]. Furthermore, female gender is a common risk factor for MC and CD, as in other autoimmune diseases. Moreover, smoking is a demonstrated risk factor for MC, considering that former and especially current smoking increased the risk for both LC and CC [2]. However, there is insufficient evidence to assess the impact of smoking cessation on the course of MC. On the other hand, a significantly reduced risk of CD among current smokers compared with never-smokers was observed in a meta-analysis published in 2018 [19]. Moreover, sustained smoking during pregnancy was inversely associated with CD in a Norwegian mother and child cohort but not confirmed in another children cohort [20]. Otherwise, in patients with CD, chronic or frequent use of some drugs (proton pump inhibitors (PPIs), nonsteroidal anti-inflammatory drugs (NSAIDs) or selective serotonin reuptake inhibitors (SSRIs)) is associated with an increased risk of developing MC, without establishing any causal relationship [1]. In CD, the duration of breastfeeding and/or timing of gluten introduction have no effect on the risk of developing the disease, as previously suggested [2]. 

First-degree relatives (5–10%), but not second-degree relatives, have been found to have a much higher risk of CD [2,21], while twin studies have shown significantly higher concordance for CD in monozygotic than in dizygotic twins (70% vs. 9%); otherwise, genetic predisposition in MC has been scarcely investigated. In fact, classic twin studies or large population-based studies are lacking, but patients with CC were more likely to report having a first-degree relative with MC than controls (OR: 10.3; 95%CI: 2.1–50.4, *p* = 0.004) [22]. 

Finally, the risk of developing CD or MC is associated with gastrointestinal infections. In fact, the first nationwide cohort study showed a significant association between gastrointestinal infections and the risk of MC, which was stronger for the CC subtype (aOR: 3.23, 95% CI: 2.81–3.7) compared to LC (aOR: 2.51, 95% CI: 2.28–2.76, *p* = 0.005) [23]. In particular, *Clostridioides difficile*, *Norovirus* and *Escherichia* species have all been associated with an increased risk of developing MC [22]. In CD, the risk of developing the disease increased synergistically in infants with a history of repeated infections, specifically implicating *Rotavirus* infection [2,24,25]. In addition, children with an increased genetic risk of CD had a higher frequency of Enteroviruses in their stool samples before the onset of CD than healthy controls [26]. It is likely that, in both diseases, gastrointestinal infections, possibly by altering the gut microenvironment, can lead to T cell activation via MHC molecules. However, the real mechanism still remains unclear. 

### 4.2. Clinical Symptoms

Both disorders share some clinical symptoms, but there are several differences in the clinical expression for CD and MC diseases. Chronic watery non-bloody diarrhea is a shared symptom of both entities. In fact, it is the most common symptom in MC, which is frequently associated with other concomitant intestinal symptoms as well as fecal urgency (55%), nocturnal stools (35.3%) and fecal incontinence (26.3%). Less frequent symptoms, with varying prevalence among studies, were described in MC patients (abdominal pain, weight loss and bloating) [1]. However, while the clinical expression of MC is quite homogeneous, in patients with CD, the symptoms can vary from the typical gastrointestinal symptoms associated with overt malabsorption to extra-intestinal manifestations (late puberty, short stature, fatigue, dermatitis herpetiformis, etc.) or nutritional deficiencies (iron deficiency anemia) associated with a substantial reduction in the intestinal absorptive surface area due to intestinal villous atrophy [2]. In fact, in a significant number of cases, CD may present with extra-intestinal symptoms (dermatitis herpetiformis, neurological symptoms such as ataxia, etc.) or signs and may be asymptomatic [2], unlike MC patients.

### 4.3. Common Autoimmunity Background

Autoimmunity is a shared characteristic between MC and patients with CD, but, while the role of autoimmunity in CD is well-established, its role in MC is still under investigation. In fact, CD is over-represented in patients with other autoimmune diseases such as type I diabetes, autoimmune liver disease, autoimmune thyroid disease and chromosome abnormalities such as Down and Turner syndromes [2]. Furthermore, other autoimmune phenomena are observed in CD, as well as the presence of high titers of autoantibodies to tissue transglutaminase 2 (TG2) in patient sera, which is now the most widely used serological test for the diagnosis and follow-up of patients with CD [2,27]. 

In contrast, in MC, there is no direct evidence to date that autoimmunity is a key mechanism in the pathogenesis of MC. However, its role is suggested by the association of MC with other diseases involving immune dysregulation that share common human leukocyte antigen (HLA) haplotypes, the presence of autoantibodies in a subset of patients, the predominance of MC in older women who are more susceptible to autoimmune processes and its ability to respond to corticosteroids therapy [5]. In fact, one hypothesis for the pathogenesis of MC is an autoimmune response triggered by an unidentified luminal antigen from the ileal stream. However, few studies have evaluated the prevalence of autoantibodies in MC, observing an increasing level of anti-nuclear antibodies (ANAs) and anti-Saccharomyces cerevisiae antibodies (ASCAs) in CC [5,28]. Another study has observed an increased prevalence of ANAs, ASCA IgG, p-ANCAs, Anti-TPO and Anti-GAD antibodies in patients with MC compared to the general population [29]. However, the difference is small and will not be observed in a large number of patients. There was no difference between the prevalence of autoantibodies in CC and LC patients. Therefore, a useful clinical marker for MC has not yet been identified. Moreover, MC patients are more likely to have concomitant autoimmune diseases that could be diagnosed in up to 50% of MC patients, being more prevalent in CC than LC patients [5]. The autoimmune diseases more commonly (1/100 to 1/10) observed in CC and LC are as follows: CD, autoimmune thyroid disease, type I diabetes mellitus, rheumatoid arthritis, seronegative polyarthritis, systemic or cutaneous lupus erythematosus, CREST syndrome/systemic sclerosis/scleroderma, ankylosing spondylitis/spondyloarthropathy, Sjögren’s syndrome and psoriasis/psoriatic arthritis among others [5]. However, it is not known whether the association of autoimmune diseases with CC and LC is due to an underlying autoimmune disease affecting both the colon and other organs, or whether the increased intestinal bowel permeability due to epithelial barrier dysfunction allows antigens to cause cross-reactivity.

### 4.4. Common Functional Bowel Disorders Overlapping

CD as well as MC can overlap with multiple other gastrointestinal diseases, including functional bowel disorders with a predominance of diarrhea (diarrhea-predominant irritable bowel syndrome (IBS-D) and functional chronic diarrhea (FDr)), among others. It is important to highlight that the functional bowel disorders with a predominance of diarrhea are very frequent disorders with a pooled prevalence of 1.4% (95%CI: 0.9–1.9) for IBS-D and 4.7% (4.5–4.9) for FDr [30]. Differential or concomitant diagnosis is important to properly manage CD and MC patients, considering the different treatment strategy needed. In particular, the prevalence of CD or MC in patients with functional bowel disorders with a predominance of diarrhea varies across studies and geographical regions, considering that many patients can develop IBS-D or FDr symptoms during the follow-up. 

Considering that patients with IBS-D or FDr are an at-risk group for CD with an expected prevalence ranging from 2.1 to 5.2%, recent international guidelines suggest that CD could be excluded in all the functional bowel disorders associated with diarrhea by the determination of IgA class anti-tissue transglutaminase antibodies by an enzyme-linked immunosorbent assay while consuming a gluten-containing diet [30]. Similarly, in a meta-analysis including studies with patients meeting the criteria for IBS-D, the prevalence of MC was 9.8% (95% CI: 4.4–17.1) [31]. Therefore, recent international guidelines suggest that MC could also be excluded in all the patients undergoing a colonoscopy for suspected IBS-D or FDr who take multiple mucosal biopsies of the colon [30].

### 4.5. The Different Gluten Effect in Patients with MC

Gluten is the water-insoluble protein mass that is left over from the washing of wheat dough to remove starch, albumins and other water-soluble proteins [2]. In CD, gluten exposure is essential for the disease development. In fact, the mainstay of the treatment in these patients is strict lifelong adherence to a GFD, which induces symptom improvement, the reversal of small-bowel villous atrophy and the normalization of associated serum antibody levels [2].

However, the role of gluten in MC has not been studied in depth. A previous study described how a gluten enema can induce LC-like changes in patients with CD [32], and several case studies have shown that patients with coexisting MC and CD may respond to a GFD [16,33,34]. However, a 2019 prospective cohort study reported no association between gluten intake and the risk of MC in two large prospective cohorts of US women (the Nurses’ Health Study (NHS) and NHSII) without CD [35]. Moreover, in another study, high intakes of dietary gluten for more than one month in two patients with established LC were not associated with histological changes in the proximal small intestine or changes in the severity of inflammation in the colon [36]. Finally, in a large series of established CD diagnoses (n = 1009), the majority of the MC cases occurred after the diagnosis of CD in patients who had already started a GFD [13]. Furthermore, no clear correlation was observed between the changes in duodenal and colonic biopsies following dietary changes, suggesting that, even in patients with CD, the adherence to a GFD may not alter the development or progression of MC [13]. 

These data suggest that the pathogenesis of MC is unlikely to be related to gluten consumption, and, unlike CD, it is unlikely that gluten withdrawal would lead to improvement in histological inflammation or clinical symptoms. However, the question of whether gluten intake may play a role in a sub-group of cases with a specific genetic susceptibility has not yet been properly ruled out. 

## 5. Common Ground in Pathogenetic Mechanisms between CD and MC

**Figure 1:**
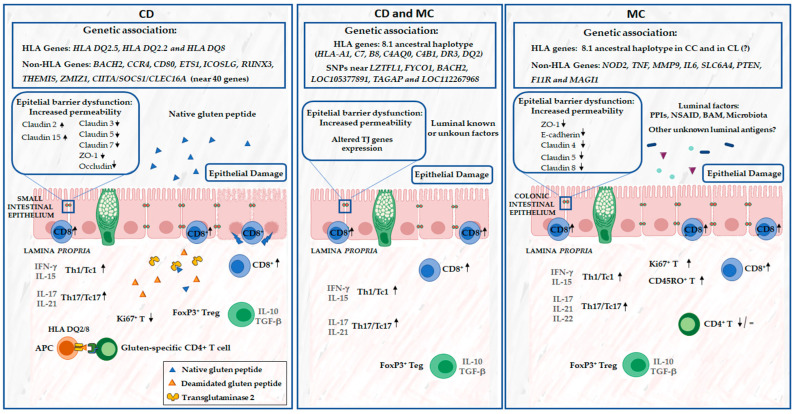
Genetic susceptibility, epithelial barrier dysfunction and adaptative immunity in both celiac disease (CD) and microscopic colitis (MC) intestinal mucosa. ↑ increased expression and/or cell population; ↓ decreased expression and/or cell population.

CD and MC share more than a simple epidemiological association; in fact, both disorders seem to be multifactorial diseases involving innate and adaptive immune responses to known or unknown luminal factors based on a rather common genetic ground (Figure 1). 

### 5.1. Genetic Factors

The HLA region, located on the short arm of chromosome 6, is the major genetic predisposing factor for CD [37]. This region contains hundreds of genes with immunological functions associated not only with CD but also with other immune-mediated diseases. In particular, 90% of the patients with CD carry the HLA-DQ haplotype HLA-DQ2.5 (encoded by *DQA1*∗*05* and *DQB1*∗*02*), while the remaining 10% of patients carry HLA-DQ2.2 (encoded by *DQA1*∗*02:01* and *DQB1*∗*02*) or HLA-DQ8 (encoded by *DQA1*∗*03* and *DQB1*∗*03:02*), with an approximately equal distribution between the two haplotypes [37]. It is important to note that, in Western countries, 40% of the general population have one or both of these variants, but the majority do not develop CD [38]. Furthermore, several non-HLA genes encoding the immune molecules involved in the function of T cells and B cells as well as in the regulation of epithelial cell polarity have been identified in CD [39,40,41]. 

While the genetic susceptibility to CD has been studied extensively, little research has been conducted on MC (Table 1 and Table 2). 

A genome-wide association study (GWAS) has demonstrated the candidate genes of CD associated with MC [50]. The SNPs across celiac associated regions on chromosomes 3p21.31, 6q15, 6q25.3 and 1q24.3 were significantly associated with MC [50]. In particular, some of these SNPs are in loci associated with immunological function as protein trafficking to the ciliary membrane, microtubule transport of autophagosomes and transcription regulator proteins with a role in the regulation of B cells and proteins involved in T cell activation. In addition, there are a few SNPs in regions of the genome with unknown functions [50]. However, some of these genes link CD to MC and are candidates to influence the pathogenesis of both diseases (Table 3). 

Moreover, other non-HLA candidate genes have been studied in MC, including nucleotide-binding oligomerization domain-containing protein 2 (NOD2), tumor necrosis factor (TF), matrix metallopeptidase 9 (MMP-9), interleukin 6 (IL6), polymorphism 5-HTTLPR in the serotonin transporter solute carrier family 6 member 4 (SLC6A4) gene and phosphatase and tensin homologue (PTEN) [42,43,44,48]. 

While some non-HLA candidate genes have been described in MC, several studies have demonstrated the role of the HLA region and the extended 8.1 haplotype in the pathogenesis of MC, particularly in CC, albeit with controversial results [16,45,46,47,51,52,53]. In fact, this haplotype has been shown to confer risk for both CD and MC, covering many allele combinations (HLA-A1, C7, B8, C4AQ0, C4B1, DR3 and DQ2) associated with immunopathological diseases [45,46]. However, while some studies have found an unequivocal association of the HLA 8.1 haplotype only for CC, others have found an association with both MC subtypes [16,45,46,52,53]. This may be due to the heterogeneity of the techniques used to analyze the genetic variants or to population bias with a lack of clinical information. Of note, the latest first genome-wide analysis of the genetic susceptibility factors for CC and LC, using large cohorts from Europe and the USA, has recently been published [54]. A strong HLA association with CC with the documented exclusion of CD diagnosis was confirmed, with the DRB1*03.01 allele and its residues Y26, N77 and R74 being key to this association [54]. However, no HLA signal was detected for LC patients, suggesting that this region is probably not relevant to LC pathogenesis [54]. Therefore, HLA associations may be CC-specific, considering that they were not detected in an LC cohort of patients with adequate statistical power, making it necessary to expand our knowledge to provide a future genotype-driven CC and LC patient stratification. 

After adjusting for the 8.1 haplotype, the primary CD risk alleles HLA-DQA1*05:01/HLA-DQB1*02:01 were no longer associated with the risk of CC, indicating that different biological pathways may be involved in CC pathogenesis [52]. Furthermore, in CC, an independent protective effect of an HLA class II-related allele (HLA-DRB1*04:01) has been shown to be a risk factor for CD [52]. Furthermore, in order to find the shared genetic effects between CC and CD, among other diseases, ASSET followed by CPGayes was performed, observing four pleiotropic signals shared with CD as well as other signals with other immune-mediated disorders, such as inflammatory bowel disease (IBD) [52]. Further research is necessary to elucidate the complex mechanisms by which HLA alleles influence the risk and protection associated with the development of CD and MC. 

### 5.2. Impairment of Intestinal Epithelial Barrier Function in CD and MC 

Alterations in intestinal permeability can lead to the increased uptake of luminal antigens, which has been implicated in several intestinal diseases, including CD, MC, inflammatory bowel disease and IBS, as well as extraintestinal diseases. 

CD is known to result from alterations in the intestinal epithelial barrier function and the activation of innate and adaptive immune responses, leading to villous atrophy and crypt hyperplasia in the small-bowel mucosa. Increased intestinal permeability is much more common in first-degree relatives of patients with CD than in the general population, which may indicate a predisposition to develop CD in these individuals [55]. In fact, several in vitro and in vivo studies have shown the increased permeability of the small intestine and altered junctional structure between epithelial cells, leading to impaired barrier function in patients with CD [56,57,58]. Furthermore, the increased permeability also facilitates the entry of gliadin peptides into the lamina propria, crossing the epithelial layers either paracellularly or transcellularly, and these peptides can induce a potent immune response [58,59]. Moreover, gliadin can directly alter several mechanisms regulating the epithelial barrier function. Among these mechanisms, a reduction in the transepithelial resistance, an increase in the permeability to small molecules, a reorganization of actin filaments and an impaired expression of the tight junction proteins (occludin, claudin-3 and claudin-4 and the tight junction (TJ)-associated protein ZO-1 and the adherens junction protein E-cadherin) have been observed [60,61]. In fact, in active CD, the upregulation of claudin-2 and claudin-15 and downregulation of claudin-3, claudin-5 and claudin-7 and occludin and ZO-1 proteins expression were found [62]. Moreover, pro-inflammatory cytokines that are increased in CD, such as IFN-γ and TNF-α, may act synergistically to affect the TJ barrier in the gut and increase the intestinal permeability [63,64]. 

As in CD, we also observe in MC an impairment of the intestinal epithelial barrier function (Figure 1). However, there is no known luminal antigen in MC capable of triggering the inflammatory response in these patients, such as gliadin for CD. In fact, Ussing chamber experiments with colonic biopsies have been performed, showing an increased transepithelial resistance indicative of increased permeability in active CC patients compared to remission and control groups [49,65]. Furthermore, the uptake of chemically killed *Escherichia coli* K12 was found to be increased both during active disease and in remission in patients with CC [49]. Only colonic permeability seems to be increased, and small-bowel permeability impairment was observed in MC patients [66]. Moreover, decreased expression of the TJ proteins, such as E-cadherin, zonula occludens-1 [ZO-1], [67] occludin and claudin-4 [65,68], was observed in CC and LC patients. Moreover, LC patients showed a downregulation of claudin 5 and claudin 8 [65]. Noren E. et al. [69] also found an association between the decreased expression of PTEN and MAGI1 in CC and LC, respectively. All these changes lead to an impaired barrier function with the increased probability of the influx of luminal antigens (microorganisms or harmful agents, etc.) into the mucosa. However, further research is needed to elucidate the link between the role of luminal gastrointestinal factors and the alteration of the epithelial barrier function in MC subtypes.

### 5.3. Mucosal Immunity Response

Several studies have shown that an adaptive immune system is involved in the pathogenesis of CD and MC. In fact, both are immune-mediated diseases and share pathophysiological links, such as the Th1-mediated response, and a rather similar mucosal cytokine profile, predominantly with elevated pro-inflammatory cytokines such as interferon-gamma (INF-γ) [67] (Figure 1).

In both diseases, luminal antigens seem to be important in developing the inflammatory response in MC and CD. In fact, in CD, the adaptative immune response targets dietary gluten proteins. The tTG2-deamidated gliadin peptides increased their affinity to HLA-DQ2/DQ8 expressed on antigen-presenting cells (APCs). Consequently, the APCs present in the intestinal mucosa deamidate immunogenic gliadin peptides to CD4^+^T cells and activate them, leading to the activation of the adaptive immune response and production of pro-inflammatory cytokines such as INF-γ [27]. However, in MC, although the exact etiology remains unclear, an aberrant immune response to one or more non-identified luminal factors in susceptible individuals is the most accepted hypothesis [5]. This is also supported by the fact that the diversion of fecal flow by ileostomy was shown to reduce the characteristic histopathological changes in CC, which recurred after ileostomy closure [70]. Considering the leukocytes’ infiltration, an increased infiltration of the inflammatory leukocytes was observed in both the epithelium and lamina propria of patients with CD and MC. 

In fact, in the epithelium, there are increased numbers of intraepithelial lymphocytes (IELs), predominantly CD3^+^ and CD8^+^, in both disorders. However, while in CD there is an increased infiltration of TCRγδ^+^ IELs, on the contrary, in LC, TCRαβ^+^ IELs were the most frequent intraepithelial lymphocytes [71,72]. It is important to highlight that, when patients with CD introduce a gluten elimination diet, the infiltration of IELs generally decreases; however, the increased number of TCRγδ^+^ T cells persists longer than TCRαβ ^+^CD8^+^ T despite the remission of the disease via a GFD [73]. Moreover, the concurrent increase in the TCRγδ^+^ IEL counts and decrease in surface CD3^−^ IEL, known as the celiac lymphogram, has actually been shown to be a new diagnostic test for distinguishing the features in seronegative, low-grade enteropathy and potential CD, as well as in most patients with CD adhering to a GFD [74]. Furthermore, in active CD, the TCRαβ^+^CD8^+^ T cells increase their expression of NKG2D and CD94/NKG2C (activating NK receptors) and decrease their expression of NKG2A (inhibitory receptor). In parallel, enterocytes have upregulated the expression of MICA/B and HLA-E. These are recognized by the NKG2D and CD94/NKG2C receptors, respectively, suggesting that the engagement of these NK cell receptors is responsible for the killing of enterocytes [75,76,77]. As previously mentioned, MC patients also showed an increased number of colonic CD3^+^ IEL in the colonic epithelium as well as in the terminal ileum, being higher in LC than in CC [5]. These cells were predominantly CD8^+^ with the conventional TCRαβ^+^CD8αβ^+^ phenotype [5]. In addition, both CC and LC patients had higher proportions of proliferating Ki67^+^CD8^+^IELs and CD45RO^+^CD8^+^IELs. These proportions were higher in CC than in LC [5,77], and, with regard to CD4^+^ IELs, LC patients showed a decreased proportion of CD4^+^ IELs [5,78]. The expression profile of TCRγδ^+^ and TCRαβ^+^ T cells in patients with MC and concomitant CD remains unknown. 

Lamina propria lymphocytes also play a key role in both diseases. Indeed, CD is a T cell-mediated immune disease in which gluten-derived peptides activate the CD4^+^ T cells of the lamina propria. In active CD, there is marked infiltration of TCRαβ^+^ T cells in the lamina propria. During active CD, both the CD4^+^ and the CD8^+^ T cells in the lamina propria lack the proliferation marker Ki67 [79]. Otherwise, an increased proportion of CD8^+^ but reduced or unchanged proportions of CD4^+^ T cells have been described in the lamina propria of MC patients [78,80]. In fact, both CC and LC patients had elevated proportions of CD4^+^CD8^+^ LPLs, albeit only significant in CC. The CD and MC patients otherwise showed an elevated proportion of proliferating Ki67^+^ T cells and CD45RO T cells [5,78]. 

In CD as well as in MC, the pro-inflammatory response is accompanied by high levels of the anti-inflammatory cytokines IL-10 and transforming growth factor-β (TGF-β) in the intestinal mucosa [81,82,83]. This seemingly paradoxical milieu of both pro-inflammatory and suppressive cytokines suggests that regulatory mechanisms may be at work to counterbalance the abnormal immune activation induced by gliadin in CD [84]. There are subsets of CD4^+^ T cells with suppressor functions (known as type 1 regulatory T cells (Tr1) and Foxp3^+^ Tregs) in the intestines of patients with CD, which, through the release of both IL-10 and TGF-β, inhibit the pathogenic response to in vitro gliadin challenge [85,86]. However, it remains controversial whether regulatory CD4^+^ T cells are involved in causing CD. In fact, no consensus exists as to which type of regulatory CD4^+^ T cells could have a role in the disease [85,87]. In MC, CD25^+^FoxP3^+^cells were found in the lamina propria of 63% of LC and 70% of CC patients [5]. CC and LC showed an increased percentage of Foxp3^+^ cells in the lamina propria, with the most prominent increase in LC patients [5,81,88]. In fact, Carrasco A. et al. [88] showed an increased percentage of Treg (CD4^+^CD25^+^FOXP3^+^) in CC and LC, associated with higher levels of IL-10. The increased amount of regulatory Foxp3^+^ cells could be interpreted as an attempt to alleviate the ongoing inflammation. 

Furthermore, IL-17 is highly produced in the inflamed intestines of patients with CD [89], confirming the involvement of Th17 cells in the pathogenesis of CD. In fact, the T cells activated by gluten release IFN-γ, interleukins IL-21 and IL-17 and these substances trigger mucosal inflammation and directly harm the epithelial cells, ultimately leading to villous atrophy in the small intestine [90]. Moreover, gluten peptides have the ability to interact directly with epithelial cells, stimulating the production of pro-inflammatory cytokines. In particular, IL-15 plays a crucial role in enhancing the cytolytic activity of IELs, contributing to the innate cytotoxic damage of epithelial cells and increasing the intestinal permeability to luminal macromolecules, including gluten peptides [91]. In another study, the MC patients also showed a marked increase in IL-15 expression [67]. Carrrasco A. et al. [88] showed that the Th1 and Th17 cells in the colonic mucosa were lower in both CC and LC, although the gene expression of the Th1/Th17 cytokines was higher in both. In fact, other MC studies have also shown an abundance of cytokines and transcription factors linked to the Th1 and Th17 (or CD8^+^ Tc1 and Tc17) immune responses [5]. Moreover, the levels of IFN-γ, IL-12, IL-1β, IL-6, IL-17A, IL-21, IL-22, IL-23 and TNFα were increased in the inflamed mucosa of MC patients, indicating a diverse mucosal cytokine profile involving mixed Th17/Tc17 and Th1/Tc1 responses [92]. Furthermore, Tagkalidis P.P. et al. [67] also identified a Th1 mucosal cytokine profile characterized by the upregulation of IFN-γ, TNFα and IL-15 as predominant cytokines. In MC patients, there was a mucosal cytokine profile where IFN-γ was predominantly upregulated. This coincided with the induction of nitric oxide synthase and the downregulation of IFN-γ-related cell junction proteins [5], a pattern similar to that observed in CD, suggesting that it may represent a response to unknown luminal antigens.

## 6. Diagnostic Work-Up and Practical Recommendation

Considering the available data on the higher prevalence of CD in MC patients and vice versa, the guidelines of MC management and guidelines on CD management recommend to screen for CD at the work-up for MC and vice versa, especially in non-responder cases, to drive the proper treatment management of these patients. 

In particular, refractory CD is defined by ongoing symptoms of malabsorption and villous atrophy despite strict adherence to a GFD for at least 12 months and could occur in 1% of patients with CD [34]. In these patients, the initial diagnosis of CD should first be confirmed, as well as the adherence to GFD; however, the other causes of refractory diarrhea, such as MC, should be excluded, considering that another disease entity could also be present concurrently. In fact, an untreated MC could affect the quality of life of these patients unable to achieve the remission of chronic diarrhea without a specific pharmacological treatment (oral budesonide as a first-line treatment). Conversely, the international guidelines recommend screening for CD in patients with MC, particularly when a patient diagnosed with MC does not respond to the recommended first-line treatment [1,28]. 

In conclusion, clinicians should prioritize checking for MC in refractory patients with CD and vice versa. In fact, patients initially diagnosed with either CD or MC may indeed have an underlying simultaneous condition causing “refractory or recurrent” chronic diarrhea, necessitating a different therapeutic approach to achieve clinical remission. 

## 7. Conclusions

CD is the most prevalent autoimmune disorder found alongside MC. There exists a mutually influential relationship between these two conditions. The patients with CD and concomitant MC are typically older and often demonstrate more pronounced duodenal mucosal atrophy. However, MC patients presenting with concomitant CD are younger than those without comorbidities. Future studies are expected to better clarify the epidemiological association between CD and MC in non-refractory cases as well as the role of a GFD in MC patients with CD-specific genetic susceptibility. Although the link between MC and CD is well-documented, the precise pathophysiological mechanism driving this association remains unclear. Despite CD and CM being two different entities, they are multifactorial diseases in which genetic, environmental and immunological components shape their pathogenesis. Further research is needed to investigate the contribution of TCRγδ^+^ IEL to the pathogenesis of CD and concomitant MC, considering that their immunophenotyping in different segments of the intestine by flow cytometry may be useful as a differential diagnostic tool in clinical practice. Genetic susceptibility is thought to interact with known or unknown luminal factors and impaired epithelial barrier function by triggering an abnormal immune response, predominantly Th1-mediated. More studies are therefore needed to better understand the complex mechanisms involving the common pathogenetic ground contributing to the epidemiological association between CD and MC.

## Figures and Tables

**Table 1:** HLA genetic variations associated with microscopic colitis.

Gene (Chromosome)	Genetic Variants	Study Design	Study Cohort	Gene Function	Reference
***HLA*** (6p)	**(LC)** **HLA-A1 frequency**: 0.666 (66.6%) part of 8.1 ancestral haplotype **HLA-A3 frequency**: 0.00 (0%) **(CC)** **HLA-A1** (part of 8.1 ancestral haplotype) and **HLA-A3** frequencies with no significant difference	Genetic Association Study	LC n = 24, CC n = 47, controls n = 3.942	Antigen presentation	[42]
	**HLA-DQ2 frequency**: 0.64 (64%) part of 8.1 ancestral haplotype **HLA-DQ1,3 frequency**: DQ1,7 0.47 (47%); DQ1,8 0.3 (30%); DQ1,9 0.2 (20%)	Genetic Association study	MC n = 53, controls n = 429		[43]
	**(CC)** **HLA-DQ2 frequency**: 0.480 (48%) part of 8.1 ancestral haplotype	Genetic Association Study	LC n = 25, CC n = 34, controls n = 70		[16]
	**(CC)** **HLA DR3-DQ2 frequency**: 0.438 (43.8%) part of 8.1 ancestral haplotype **HLA DR4-DQ8 frequency**: 0.138 (13.8%)	Genetic Association Study	MC n = 80 (CC n = 29, LC = 51), controls n = 3.627		[44]
	**(CC)** 8.1 ancestral haplotype	Genetic Association Study	LC n = 116, controls n = 1.995		[45]
	**(CC)** 8.1 ancestral haplotype related HLA class I (A*01:01, B*08:01, C*07:01) and class II (DRB1*03:01, DQA1*05:01, DQB1*02:01)	Genetic Association Study	CC n = 1.051, controls n = 27.101		[46]
	**(CC)** 8.1 ancestral haplotype DRB1*03:01: strongest association **(LC)** no GWAS-significant signal detected for LC	GWAS meta-analyses	Europe and USA LC n = 373, CC n = 1.498, controls n = 13.487; UK Biobank and FinnGen MC n = 2.599, controls n = 552.343		[47]

HLA = human leukocyte antigen; CC = collagenous colitis; LC = lymphocytic colitis; MC = microscopic colitis; GWAS = genome-wide association study.

**Table 2:** Non-HLA genetic variations associated with microscopic colitis.

Gene (Chromosome)	Genetic Variants	Study Design	Study Cohort	Gene Function	Reference
***NOD2*** (16q12.1)	**(CC)** **NOD2 allele (carriage frequency)**: SNP 8 (9.5%), SNP 12 (1.3%), SNP 13 (8.1%)	Genetic Association Study	CC n = 75, controls n = 534	Immune response to intracellular bacterial lipopolysaccharides (LPS)	[43]
***TNF*** (6p21.33)	**TNFα, genotype (carriage frequency)**: 1.1 (53.8%); 1.2 (39.7%); 2.2 (6.4%) **TNF-2 allele carriage frequency**: 46.2%	Genetic Association Study	MC n = 78, controls n = 178	Multifunctional pro-inflammatory cytokine	[44]
***MMP9*** (20q13.12)	**(CC)** **TNF-2 allele carriage frequency**: 24%	Genetic Association Study	CC n = 75, controls n = 334	Breakdown of extracellular matrix	[44]
***IL6*** (7p15.3)	**Polymorphism IL-6-174**: Susceptibility allele GG	Genetic Association Study	MC n = 81, controls n = 178	Inflammation and maturation of B cells	[42]
***SLC6A4*** (17q11.2)	**Polymorphism 5-HTTLPR (SS) frequency**: 12% [lower than control (30%)]	Genetic Association Study	MC n = 41, controls n = 100	Serotonin transporter	[48]
***PTEN*** (10q23.31)	**rs1234224**: susceptibility allele G	Genetic Association Study	MC n = 25 (CC n = 10, LC n = 14), controls n = 58	Tight junction	[49]
***F11R*** (1q23.3)	**(CC)** **rs790055**: susceptibility allele G			Tight junction	
**MAGI1** (3p14.1)	**rs17417230**: susceptibility allele C			Tight junctions, scaffolding protein at cell–cell junctions	
**CLEC16A** (16p13.13)	**rs35099084**: susceptibility allele C	GWAS meta-analyses	Europe and USA LC n = 373, CC n = 1.498, controls n = 13.487; UK Biobank and FinnGen MC n = 2.599, controls n = 552.343	Membrane-associated endosomal protein	[47]
**RMI2** (16p13.13)				DNA repair and genome stability	

NOD2 = nucleotide binding oligomerization domain-containing 2; TNF = tumor necrosis factor; MMP9 = matrix metallopeptidase 9; IL6 = interleukin 6; SLC6A4 = solute carrier family 6 member 4; PTEN = phosphatase and tensin homolog; F11R = F11 receptor; MAGI1 = membrane-associated guanylate kinase, WW and PDZ domain-containing 1; CLEC16A= C-Type Lectin Domain Containing 16A; RMI2 = RecQ-mediated genome instability 2; CC = collagenous colitis; LC = lymphocytic colitis, MC = microscopic colitis; GWAS = genome-wide association study.

**Table 3:** Genetic variations associated with celiac disease and microscopic colitis.

Gene (Chromosome)	Genetic Variants	Study Design	Study Cohort	Gene Function	Reference
***HLA*** (6p)	**HLA-DQ1,3:** DQ1,7; DQ1,8; DQ1,9 **HLA-DQ2** Part of 8.1 ancestral haplotype	Genetic Association Study	MC n = 53, CD n = 25, controls n = 429	Antigen presentation	[43]
	**HLA-DR3-DQ2 frequency**: 13 (86.7%). Part of 8.1 ancestral haplotype **HLA DR4-DQ8 frequency**: 1 (6.7%) **HLA-DR3-DQ2 and/or** **HLA-DR4-DQ8 frequency:** 14 (93.3%)	Genetic Association Study	MC with CD n = 15, controls n = 3627		[44]
	HLA-DRB1*04:01: CC protective alleles and strong risk for CD	Genetic Association Study	CC patients n = 1051 and controls n = 27,101		[46]
***LZTFL1*** (3p21.31)	**rs4683148 frequency**: 0.39 (39%)	GWAS	MC cases n = 69 (with concomitant celiac disease), CD cases n = 1550 of North American and controls n = 3084	Protein trafficking	[50]
***FYCO1*** (3p21.31)	**rs1072755 frequency**: 0.39 (39%) **rs4535265 frequency**: 0.39(39%) **rs2234358 frequency**: 0.49(49%) **rs3796375 frequency**: 0.42 (42%) **rs737452 frequency**: 0.42 (42%)			Microtubule transport of autophagosomes	
***None reported*** (3p21.31)	**rs2373154 frequency:** 0.43 (43%)			Not described	
***BACH2*** (6q15)	**rs207270 frequency**: 0.46 (46%) **rs4142967 frequency**: 0.46 (46%) **rs12212193 frequency:** 0.46 (46%)			Adaptive immune response of T and B cell	
***LOC105377891*** (6q15)	**rs285640 frequency**: 0.33 (33%) **rs1847473 frequency**: 0.27 (27%)			RNA gene, ncRNA class	
***TAGAP*** (6q25.3)	**rs1738074 frequency**: 0.43 (43%)			T cell activation	
***LOC112267968*** 6q25.3	**rs1738074 frequency**: 0.43 (43%)			Not described	
***None reported*** (1q24.3)	**rs2227203 frequency**: 0.46 (46%)			-	

*HLA* = human leukocyte antigen; *LZTFL1 =* leucine zipper transcription factor like 1; *FYCO1* = FYVE and coiled-coil domain autophagy adaptor 1; *BACH2* = BTB domain and CNC homolog 2; *LOC105377891* = uncharacterized LOC105377891; *TAGAP* = T cell activation Rho GTPase-activating protein; *LOC112267968* = uncharacterized LOC112267968; CC = collagenous colitis; MC = microscopic colitis; GWAS = genome-wide association study.

## Data Availability

There are no private databases created or analyzed for this study.
